# Detection of clinical progression through plasma ctDNA in metastatic melanoma patients: a comparison to radiological progression

**DOI:** 10.1038/s41416-021-01507-6

**Published:** 2021-08-09

**Authors:** Gabriela Marsavela, Ashleigh C. McEvoy, Michelle R. Pereira, Anna L. Reid, Zeyad Al-Ogaili, Lydia Warburton, Muhammad A. Khattak, Afaf Abed, Tarek M. Meniawy, Michael Millward, Melanie R. Ziman, Leslie Calapre, Elin S. Gray

**Affiliations:** 1 School of Medical and Health Sciences, Edith Cowan University, Joondalup, WA Australia; 2 Centre for Precision Health, Edith Cowan University, Joondalup, WA Australia; 3 Department of Molecular Imaging and Therapy Service, Fiona Stanley Hospital, Murdoch, WA Australia; 4 Department of Medical Oncology, Sir Charles Gairdner Hospital, Nedlands, WA Australia; 5 School of Medicine, University of Western Australia, Crawley, WA Australia; 6 Department of Medical Oncology, Fiona Stanley Hospital, Murdoch, WA Australia; 7 School of Biomedical Science, University of Western Australia, Crawley, WA Australia

**Keywords:** Tumour biomarkers, Biomarkers

## Abstract

**Background:**

The validity of circulating tumour DNA (ctDNA) as an indicator of disease progression compared to medical imaging in patients with metastatic melanoma requires detailed evaluation.

**Methods:**

Here, we carried out a retrospective ctDNA analysis of 108 plasma samples collected at the time of disease progression. We also analysed a validation cohort of 66 metastatic melanoma patients monitored prospectively after response to systemic therapy.

**Results:**

ctDNA was detected in 62% of patients at the time of disease progression. For 67 patients that responded to treatment, the mean ctDNA level at progressive disease was significantly higher than at the time of response (*P* < 0.0001). However, only 30 of these 67 (45%) patients had a statistically significant increase in ctDNA by Poisson test. A validation cohort of 66 metastatic melanoma patients monitored prospectively indicated a 56% detection rate of ctDNA at progression, with only two cases showing increased ctDNA prior to radiological progression. Finally, a correlation between ctDNA levels and metabolic tumour burden was only observed in treatment naïve patients but not at the time of progression in a subgroup of patients failing BRAF inhibition (*N* = 15).

**Conclusions:**

These results highlight the low efficacy of ctDNA to detect disease progression in melanoma when compared mainly to standard positron emission tomography imaging.

## Introduction

Metastatic melanoma is the most aggressive type of skin cancer and is responsible for most skin cancer-related deaths [1, 2]. In the past decade, the clinical management of patients with advanced melanoma was revolutionised by the use of anti-programmed cell death 1 (PD1) and/or anti-cytotoxic-T-lymphocyte-associated antigen 4 (CTLA-4) immunotherapies, as well as BRAF/MEK inhibiting targeted therapies [3]. These treatments have significantly increased the 5-year survival rate of melanoma patients, which is currently at 52% for the nivolumab plus ipilimumab, 44% for nivolumab alone, 26% for ipilimumab and 34% for dabrafenib/trametinib [4, 5]. Imaging scans, such as ^18^F-labelled fluorodeoxyglucose positron emission tomography (FDG-PET) or computerised tomography (CT), are currently the clinical standard for treatment monitoring, but are costly and of limited accessibility in rural areas. Nonetheless, disease progression occurs in a large proportion of patients, even after treatment cessation [6]. Thus, biomarkers that can aid clinicians in accurately detecting progressive disease in patients are critical.

Circulating tumour DNA (ctDNA) is progressively used as a marker of disease status in multiple types of cancers, including melanoma [7]. This non-invasive real-time biomarker has been demonstrated to accurately reflect pretreatment tumour burden [8, 9], provide predictive or prognostic information and is a useful monitoring tool for treatment response [10–12]. However, very few studies [13–16] have defined the clinical validity of ctDNA to effectively identify disease progression in a large cohort of melanoma patients compared to medical imaging, the current gold standard for disease monitoring.

In this study, we quantified plasma ctDNA (*N* = 108) at the time of radiological disease progression in a cohort of metastatic melanoma patients treated with systemic therapy in the context of real-world clinical practice. We further evaluated the potential of ctDNA to detect disease progression in a prospective cohort of patients (*N* = 66). Finally, we evaluated the correlation between ctDNA levels and metabolic tumour burden (MTB) prior to treatment and at the time progression in a subgroup of patients treated with BRAF/MEK inhibitors (*N* = 15).

## Materials and methods

### Patients

Metastatic melanoma patients undergoing systemic therapies were enrolled in the study between 2013 and 2019 at Sir Charles Gairdner Hospital (SCGH) and Fiona Stanley Hospital (FSH) in Perth, Western Australia. Written informed consent was obtained from all patients under approved Human Research Ethics Committee protocols from Edith Cowan University (Nos. 11543 and 18957) and SCGH (Nos. 2007-123 and 2013-246) in compliance with the Declaration of Helsinki. Experiments were performed according to institutional and national guidelines and regulations.

We retrospectively selected patients who had plasma samples collected close to the time of the first unequivocal radiological scan indicating disease progression (PD) after commencing therapy, i.e. either 1 week before or up to 4 weeks of PD, but without any change in treatment. Blood samples were collected in EDTA vacutainer or in Cell-Free DNA BCT® (Streck, La Vista, NE). Within 24 h of blood collection, plasma was separated by centrifugation at 300 × *g* for 20 min, followed by second centrifugation at 4700 × *g* for 10 min. Isolated plasma was stored at −80 °C until extractions were performed. A total of 108 plasma samples from 93 patients were included in this analysis. For 15 patients, samples collected at progression to first- and second-line treatment were included in the analysis. Plasma samples at the time of best overall clinical response to therapy were available for 62 of the 108 cases investigated and were compared with ctDNA levels at progressive disease.

For the prospective analysis, a total of 66 patients were enrolled and monitored for plasma ctDNA every 3–12 weeks, with a median follow-up duration of 66 weeks (range 26–110 weeks). All blood samples were collected in Cell-Free DNA BCT® (Streck) and plasma was stored at −80 °C until extractions were performed.

### ctDNA assessment

Selection of mutational targets for ctDNA analysis was identified via standard pathology protocols or using a customised melanoma next-generation sequencing (NGS) panel (Illumina, San Diego, CA, USA) as described by Calapre et al. [17], based on the following criteria: (i) known melanoma hotspot mutation in*BRAF, NRAS* and/or *TERT* promoter; (ii) COSMIC/TCGA reported melanoma-associated mutations; (iii) other mutation with a PolyPhen score > 0.7 and high variant allele frequency in the tumour. Commercially available and/or customised probes were used to analyse ctDNA by droplet digital PCR (ddPCR). A list of all mutations used for ctDNA detection is provided in Table S1. The limit of blank for each assay used for ctDNA detection was previously reported by Calapre et al. [17] and Marsavela et al. [18].

Cell-free DNA (cfDNA) was extracted from plasma using the QIAamp Circulating Nucleic Acid Kit (Qiagen, Hilden, Germany). Extracted cfDNA was then analysed by ddPCR as previously described [12]; samples that were negative in the first instance were tested further in duplicate to complete a triplicate set. A positive control, a healthy control and a no-template control were included in each run. Only tests providing >10,000 droplets were used for analysis.

### Disease progression assessment

Tumour disease progression was assessed radiologically by CT and/or FDG-PET scans. Magnetic resonance imaging (MRI) of the brain was also used where indicated. Patients were considered to have PD if they developed new lesions, had a significant increase in tumour size as per RECIST 1.1 or iRECIST on CT, had increased metabolic activity on FDG-PET scan or presented a new or enlarging clinical lesion, confirmed by the treating clinician. Clinicians were blinded to the ctDNA result at the time of the scan.

### MTB analysis

MTB was calculated from FDG-PET scans as described previously [8, 17]. All images were reviewed retrospectively by an experienced nuclear medicine physician (ZA-O) blinded to the ctDNA analysis. Total lesion glycolysis, which combines volumetric and metabolic information, was calculated for MTB evaluation [19, 20]. Analyses were conducted on a Siemens Syngo via workstation (Siemens Healthcare GMbH, Erlangen, Germany).

### Statistics

Differences between ctDNA levels were estimated by unpaired *t* test from the log-transformed data. Differences between the detection rates were assessed using two-sided Fisher’s exact test. Paired *t* test was used to evaluate the difference between ctDNA levels at the time of response and clinical progression. All variables were tested for normality and equality variance. Statistical differences between ctDNA levels at the time of response and progression in the same individual were also assessed using a Poisson distribution test, using calculated mutant DNA concentrations and the number of DNA occupied droplet counts as analytical variables [21]. Correlations between MTB and ctDNA were carried using Pearson’s correlation of the log-transformed values. Statistical analyses were performed using R version 5.2, GraphPad Prism version 5 and SPSSv22.0. Results were considered statistically significant at *P* < 0.05.

## Results

### ctDNA detection at disease progression

**Fig. 1:**
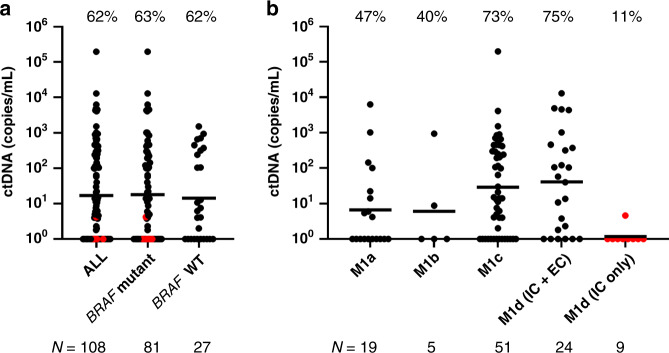
ctDNA quantification in melanoma patients at the time of progressive disease on systemic therapy. **a** Plasma ctDNA levels (copies/mL of plasma) in melanoma samples (*N* = 108), stratified by mutation status. Red dots identify patients with the intracranial disease only. **b** M1d cases were further subdivided into those with extracranial (IC + EC) and those with brain-only metastases (IC only). Percentages denote the frequency of patients with detectable ctDNA. The geometric mean of ctDNA concentrations is indicated for each group by a line.

We retrospectively selected 108 plasma samples collected at the time of disease progression to determine the rate of ctDNA detection. Patient characteristics and clinical parameters for both cohorts are summarised in Table 1. The overall detection rate was 62% and was independent of the tumour *BRAF* mutational status (Fig. 1a). When subdivided by disease stage, patients with visceral metastases at progression (M1c and M1d with extracranial metastases) had higher ctDNA levels and detection rates compared to those patients who progressed with lymph node, subcutaneous or lung lesions (Fig. 1b). Only 9 of 19 cases with metastases in the skin, subcutaneous tissue or lymph node (M1a, 47%) had detectable ctDNA at progression. In addition, only two of the five cases with lung metastases (M1b, 40%) had detectable ctDNA. Notably, all but one of the nine patients with the intracranial disease only (M1d IC only) had undetectable ctDNA at the time of progression, in contrast to the high detection rate amongst those with extracranial involvement.

**Table 1:** Clinical characteristics at baseline of the melanoma samples included in the study.

Variable	Retrospective cohort, *N* = 108 (%)	Prospective cohort, *N* = 45 (%)
Age		
≤60	54 (50)	24 (53)
>60	54 (50)	21 (47)
Gender		
Female	38 (35)	14 (31)
Male	70 (65)	31 (69)
Mutation status		
*BRAF* mutant	81 (75)	32 (71)
*NRAS* mutant	11 (10)	4 (9)
*BRAF/NRAS* WT	16 (15)	9 (20)
*Treatment*		
ICI		
Pembrolizumab	22 (20)	6 (13)
Ipilimumab/Nivolumab	15 (14)	15 (34)
Ipilimumab	11 (10)	
Targeted therapies		
Vemurafenib	3 (3)	1 (2)
Dabrafenib/Trametinib	54 (50)	16 (36)
Vemurafenib/Cobimetinib	2 (2)	4 (9)
Adjuvant		
Nivolumab	1 (1)	1 (2)
Clinical trials		2 (4)
No progressive disease	–	29 (64)
Progressive disease	108 (100)	16 (36)
Response prior to PD		
Yes	67 (62)	16 (36)
ctDNA not elevated at PD	34 (31)	7 (16)
ctDNA elevated at PD	33 (31)	9 (20)
ctDNA statistically elevated at PD	20 (19)	5 (11)
No	41 (38)	–
AJCC stage/M classification at PD		*N* = 16
M1a	19 (18)	5 (31)
M1b	5 (5)	2 (13)
M1c	51 (47)	4 (25)
M1d	33(30)	5 (31)
Brain-only metastasis at PD		
Yes	9 (8)	4 (25)
No	99 (92)	12 (75)

ICI immune checkpoint inhibitors, PD progressive disease.

**Fig. 2:**
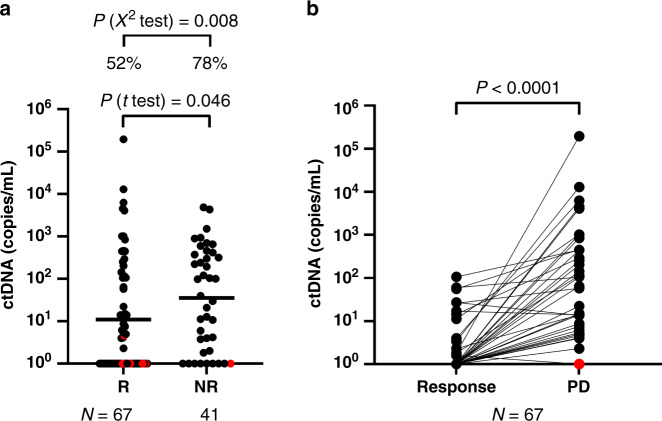
Plasma ctDNA levels are relative to progression. **a** Plasma ctDNA levels (copies/mL of plasma) in melanoma samples at the time of progression in patients who had a response to therapy (R) or did not show response to treatment (NR). The geometric mean of ctDNA concentrations is indicated for each group by a line. The *P* values of a *t* test comparing the log-transformed ctDNA levels and Fisher’s exact test comparing detection rates are indicated above each comparison. **b** Changes in ctDNA levels between the time of response and the time of progression. Percentages denote the frequency of patients with detectable ctDNA. Red dots identify patients with intracranial disease only. The geometric mean of ctDNA concentrations is indicated for each group by a line. Paired *t* test *P* value of the log-transformed ctDNA levels is indicated above the graph.

From a total of 108 cases, 67 (62%) showed a response to therapy prior to progressive disease. Interestingly, patients who responded to treatment had significantly lower ctDNA levels at disease progression (*P* = 0.046; Fig. 2a). Moreover, the detection rate was significantly lower in patients who responded (35/67, 52%) compared to those who did not respond to therapy and had no tumour size reduction (32/41, 78%, *P* = 0.008).

In this subgroup of patients with samples taken at the time of response to treatment and PD, paired comparison of ctDNA levels showed a significant increase in ctDNA at progression relative to the point of response or nadir (*N* = 67, *P* < 0.0001; Fig. 2b). Of these, 22 cases (34%) had undetectable ctDNA at the time of best response, which became positive at progression. However, only 30/67 (45%) of all cases had a significant increase in ctDNA level when ddPCR results are compared using a Poisson test.

### ctDNA detection at the time of progression in a prospective cohort

**Fig. 3:**
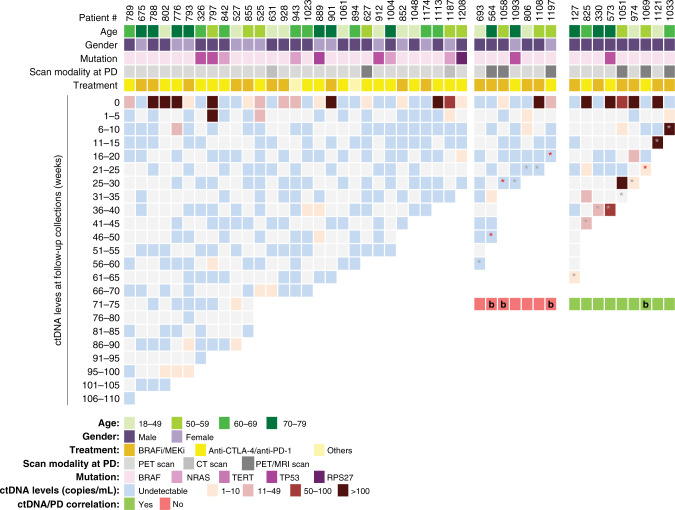
Plasma ctDNA levels in the prospective cohort during the follow-up period relative to clinical progression. Columns represent each patient’s age, gender, mutation status, scan modality used for monitoring, treatment and longitudinal quantitative ctDNA results. Columns are stratified into patients without or with disease progression, and the latter separated into those with detectable or undetectable ctDNA. b—Intracranial disease only. Grey*- Progressive disease. Red *- Presence of only intracranial malignant disease at progressive disease.

To validate these results, we prospectively recruited a total of 66 metastatic melanoma patients either commencing or undergoing systemic treatment, including two cases treated with adjuvant therapy after removal of isolated tumour metastases. Of the 64 patients with active disease, 14 did not respond to therapy and were excluded from the analysis. Of the 52 patients with objective responses (*N* = 50) or who had adjuvant therapy (*N* = 2), 23 (44%) experienced progression during the follow-up period. However, for seven cases, we did not have a blood collection for ctDNA analysis within 2 weeks of disease progression. Therefore, follow-up collections for a total of 45 patients, 16 with PD and 29 with the ongoing response, were included in the final analysis presented in Fig. 3. The clinical characteristics of these 45 patients can be found in Table 1. The majority of patients were males (101/153, 66%) and treated with combination therapy, either dabrafenib/trametinib (70/153, 46%) or ipilimumab/nivolumab (30/153, 20%). The prospective cohort largely consisted of patients who received immunotherapy and the proportion of *BRAF* mutants was lower than in the retrospective cohort.

Most patients experiencing disease progression were treated with BRAF/MEK inhibitors (11/16, 69%, Fig. 3). Amongst the 16 patients who relapsed, disease progression was detected at a median time of 31 weeks from the commencement of treatment (range 10–64 weeks). Only 9 of 16 patients had elevated ctDNA levels at the time of progression (56%; Fig. 3). From the nine patients with increased ctDNA levels at disease progression, except for case #1069, all others had a significant increase in ctDNA level when ddPCR results were compared using Poisson statistics. By contrast, seven cases remained ctDNA negative despite radiological disease progression (44%). Of these, three had intracranial disease-only progression (red asterisks; Fig. 3). Of note, one of the three patients with the intracranial disease only (#1069) developed a leptomeningeal disease and had a detectable but low plasma ctDNA (1.4 copies/mL) at progression.

Notably, four patients had no detectable plasma ctDNA at the time of progression with clear extracranial disease. One patient showed progression in the right external iliac node (#693), one had a new subcutaneous lesion on the left thigh (#1093), another developed several new bilateral pulmonary nodules at the time of progression (#806) and one (#1108) showed bone recurrence at the T2 vertebral body.

For two cases ctDNA became detectable 10–25 weeks prior to radiological disease progression (Fig. 3). Patient #974 showed partial response prior to progressive disease in lung and lymph nodes detected by PET scans. Analysis of plasma collected 24 weeks prior to progression indicated an increase in ctDNA levels, but radiological examination demonstrated no evidence of progressive disease. Similarly, patient #825 showed increasing ctDNA levels at ~13 weeks before the clinical and radiological progressive disease was identified in multiple lymph nodes, gallbladder, duodenum and subcutaneous nodules.

Patients without unequivocal disease progression (*N* = 29) were also followed up for a median of 66 weeks (range 26–110). Plasma ctDNA was undetectable in the majority of plasma samples. However, we observed that ten patients (34%) had intermittent low ctDNA levels without apparent clinical cause. Of note, ctDNA levels were always below 10 copies/mL and undetectable in later follow-up samples.

### Correlation between ctDNA levels versus MTB at baseline and disease progression

Previously, we observed that in pretreatment samples from metastatic melanoma patients, ctDNA plasma concentrations strongly correlated with MTB derived from PET scans [8]. Here, we investigated the correlation between ctDNA and MTB at the time of progression in 15 patients treated with dabrafenib plus trametinib with matching samples at baseline and progression. We selected this subgroup as they constitute the majority of patients monitored in this study who developed disease progression after an objective response during the ctDNA monitoring period.

**Fig. 4:**
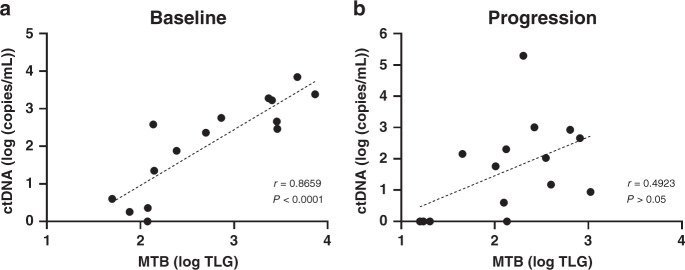
Plasma ctDNA and MTB correlation. **a**, **b** Correlation between ctDNA levels and MTB in melanoma patients treated with combination BRAF/MEKi-targeted therapies at baseline (**a**) and radiological detection of progression (**b**). Pearson’s correlation coefficient and *P* values are indicated for each comparison.

While we found a strong correlation between ctDNA and MTB at baseline (*r* = 0.8659, *P* < 0.0001), no correlation was apparent at disease progression (*r* = 0.4923, *P* > 0.05) (Fig. 4). These results suggest that ctDNA levels do not reflect disease burden and its metabolic activity at progression.

## Discussion

The high number of therapies available for late-stage melanoma necessitates an efficient stratification of patients to appropriate treatments. In addition, rigorous monitoring for timely detection of disease progression is also necessary, to ensure swift modification of treatment and improve patient outcomes. Plasma ctDNA has been heralded as an alternative pathway to monitor disease in cancer patients, and can potentially provide lead time to overt progression within which treatment could be modified [13]. Here, we made use of a real-world cohort of metastatic melanoma patients to determine the reliability of ctDNA for the detection of disease progression. Our results demonstrated that ctDNA elevation was only able to indicate the progression in 52–56% of patients, highlighting the limitations of ctDNA as a reliable marker to monitor progression alone.

We observe that low ctDNA copies (<10 copies/mL of plasma) were detected sporadically in patients who do not show radiological progression. Therefore, stringent criteria are required to indicate disease progression by ctDNA detection. We attempted to address this by conducting a Poisson test to determine statistically significant increases in ctDNA. Accordingly, only 30 of 67 (45%, retrospective cohort) and 8 of 9 (89%, prospective cohort) patients were found to have a statistically significant increase in ctDNA concentration at PD relative to the response. Overall, this reduces the rate of ctDNA detection to 47%. These results highlight the low efficacy of ctDNA to detect PD in most patients when compared to standard PET imaging.

Notably, one major challenge for the use of ctDNA as a monitoring biomarker of progressive disease is the lack of detectable tumour-derived fragments in the plasma of patients with brain metastases [22, 23]. In our study, 3 of the 7 (43%) ctDNA-negative patients in the prospective study and 8 out of 41 (20%) of ctDNA-negative cases in the retrospective study had intracranial disease only at the time of progression. Accounting for patients who had an initial response to therapy in both the retrospective and prospective cohort, a significant proportion had intracranial disease progression (12–19%). The presence of isolated brain metastases poses a significant limitation for the use of plasma ctDNA as a surveillance biomarker, considering that at the time of diagnosis of metastatic melanoma nearly 20% of patients will only have metastasis in the brain and >50% of patients undergoing targeted therapies or immunotherapies will eventually develop the progressive disease in the brain [24–26]. Thus, for these patients, plasma ctDNA is not suitable for monitoring disease progression, and MRI may remain a critical modality for monitoring disease progression in melanoma. Nonetheless, previous research [27–30] has shown that ctDNA is detectable in the cerebrospinal fluid (CSF) of cancer patients with brain metastases only. Assessment of the dynamic changes of ctDNA in CSF, but not in plasma, is potentially a valid surrogate marker in such situations.

Immunotherapy with anti-CTLA-4 or anti-PD-1 agents presents a challenge for oncologists due to pseudoprogression. This pattern is defined as initial radiographic tumour growth followed by regression and occurs in 10–15% of patients with metastatic disease [31, 32]. This delayed immune response could lead to an erroneous indication of refractory disease, which is associated with ctDNA negativity [33]. In this study, disease progression by radiological scans were all confirmed by a second scan that shows disease progression or a tumour biopsy that confirms malignancy. Based on these parameters, the lack of ctDNA at progression was therefore not attributable to potential pseudoprogression events.

Despite the strong suggestions that ctDNA may provide a lead time for detection of disease progression [9, 12–14], here we observed that ctDNA failed to successfully detect disease progression in patients that initially responded to therapy. Only a few studies have previously assessed the ctDNA detection rate at disease progression in melanoma. Rowe et al. [14] presented a detection rate of 100% in a small cohort of five patients that developed disease progression after showing response to therapy. Moreover, they reported detectable ctDNA in four of these patients prior to radiological detection of progression. However, the statistical significance of this increase is not described. Similarly, Haselmann et al. [13] has also reported a high percentage of cases with ctDNA detection prior to radiological progression (61%, *N* = 11/18). However, the prior response to therapy in these patients is unclear. In addition, from the 18 cases monitored, 14 cases had detectable ctDNA at disease progression (78%). More recently, Váraljai et al. described that ctDNA increase preceded radiologic progression with an average lead time window of 3.5 months in 86% of the 36 melanoma patients [34]. A study by Schreuer et al. described an increase in *BRAF* mutant ctDNA in patients treated with targeted therapies at the time of radiological disease progression assessed by PET scans in 36 patients. This increase was detected with a sensitivity of 70% (*N* = 19/27) and specificity of 100% [16]. Based on our results and that of others, there is currently no congruence of ctDNA detection rate at PD in melanoma, and therefore further studies are required to elucidate factors influencing these results.

The lower detection rate reported in our study, in contrast to the above-mentioned reports, may be influenced by the imaging screening modality. For most of our patients (66%), disease progression was identified by PET scans. The widespread use of PET for monitoring melanoma patients in Australia results in more sensitive surveillance of progression compared to CT scans. In a meta-analysis that pooled data from 74 studies containing 10,528 melanoma patients, PET was found to be superior to CT for the surveillance of metastatic disease [35]. This PET improved performance in depicting metastatic lesions over conventional imaging modalities; however, CT has been commonly described in the literature [36, 37].

Reconciling the findings of previous studies that used CT scan as a mode of restaging [13, 14, 34], and with our observation predominantly contrasted to PET scans, ctDNA detection rates at progression is likely to have comparable if not superior sensitivity when compared only to CT scans, given that there must be a sizable volume of disease for a significant increase to be detected. Thus, the clinical utility of ctDNA for progressive disease monitoring should be evaluated relative to the prevalent imaging modality. Despite ctDNA not showing high detection rates when compared with PET monitoring, its use may allow monitoring of patients that live in rural areas, potentially aiding adjustment of inequality of health access.

Previously, we have shown that patients with <10 MTB score, which equates to a low disease burden, have ctDNA levels that fall within the range of sampling error and therefore affect detection. In addition, despite the high correlation found previously between ctDNA levels and disease burden [8], we observed that ctDNA levels do not correlate with MTB at the time of progression in patients treated with targeted therapies. This also aligns with our previous report that ctDNA is not predictive of outcome to second-line immunotherapy following targeted therapy failure [18].

It is possible that cellular mechanisms that mediate treatment resistance may also interfere with cell apoptosis rates and ctDNA shedding. The cellular mechanism through which ctDNA is shed is poorly understood and its source has been extended to apoptotic tumour cells [38], tumour-derived extracellular vesicles [39], disseminated tumour cells and circulating tumour cells [40, 41]. However, no systematic study is yet to discover whether location within an organ, vascularisation or mitotic rate affect the amount of released ctDNA [42]. In addition, Smith et al. [43] have shown that the number of macrophages and tumour necrosis factor-α expression within the tumour is increased in patients treated with BRAF and MEK inhibitors. The increased presence of macrophages in relapsed tumours resistant to MAPK inhibitors might play an important role in clearing apoptotic cells and maintaining low ctDNA levels in the blood. Improved methods for the detection of ctDNA need to be accompanied by an improved understanding of the relationship between ctDNA release and the pathological state of the tumour.

There are a few limitations in our retrospective study. The timing of the blood collections relative to imaging scans varied between patients. Given the short half-life of ctDNA, blood drawn should ideally be conducted immediately prior to imaging to ensure that the ctDNA detected is a true representation of the lesions identified by the imaging technique. Moreover, pre-analytical factors such as plasma separation time, centrifugation speed or plasma extraction volume may also have affected our results [44].

The targeted principle of the ddPCR assay may also be a limiting factor in the detection rate of ctDNA at progression. Studies exploring the use of untargeted methods that simultaneously interrogate multiple cancer-specific variants based on the use of NGS techniques have also been shown to improve both sensitivity and specificity of ctDNA detection [45, 46]. As sequencing costs are reduced, NGS-based untargeted strategies may become clinically feasible.

In summary, our real-world cohort study highlights the urgent need to improve the methods used to detect ctDNA at disease progression and investigate the biological nature of ctDNA shedding to increase the clinical application of this non-invasive liquid biopsy. Future clinical trials with simultaneous imaging evaluations and ctDNA plasma sampling are needed to accurately define the clinical utility of ctDNA at detecting disease progression.

## Supplementary information

Supplementary Materials

Reproducibility checklist

## Data Availability

The datasets generated during the current study are available from the corresponding author on reasonable request.
