# Supplementary material for: Detection of clinical progression through plasma ctDNA in metastatic melanoma patients: a comparison to radiological progression
Source: Br J Cancer. 2021 Aug 9;126(3):401–8. doi: 10.1038/s41416-021-01507-6 (PMC8810871; doi:10.1038/s41416-021-01507-6)
Supplement: Supplementary file 1 — Supplementary Materials [file 41416_2021_1507_MOESM1_ESM.docx]

**Supplementary Materials**

**Table S1:** Mutation variants of the melanoma samples included in the study.

| ***Variable*** | ***Retrospective Cohort***  *N=108 (%)* |  | ***Prospective Cohort***  *N=45 (%)* |
| --- | --- | --- | --- |
|  |  |  |  |
| ***BRAF mutation status*** |  |  |  |
| *BRAF V600E* | *59 (55)* |  | *19 (43)* |
| *BRAF V600K* | *13 (12)* |  | *10 (23)* |
| *BRAF V600R* | *6 (5)* |  |  |
| *BRAF V600E2* | *2 (2)* |  | *2 (4)* |
| *BRAF K601E* | *1 (1)* |  | *1 (2)* |
| *BRAF D594N** | *1 (1)* |  |  |
| *BRAF E586K** | *1 (1)* |  |  |
| *BRAF G615V** | *1 (1)* |  | *1 (2)* |
| *BRAF L597Q** | *1 (1)* |  |  |
| *BRAF S467L** |  |  | *1 (2)* |
| ***NRAS mutation status*** |  |  |  |
| *NRAS Q61K* | *6 (5)* |  | *1 (2)* |
| *NRAS Q61R* | *2 (2)* |  | *2 (4)* |
| *NRAS Q61H* |  |  | *1 (2)* |
| *NRAS Q61L* | *1 (1)* |  |  |
| *NRAS G12D* | *1 (1)* |  |  |
| *NRAS G13D* | *1 (1)* |  |  |
| ***TERT mutation status*** |  |  |  |
| *TERT C228T* | *2 (2)* |  | *4 (10)* |
| *TERT C250T* | *3 (2)* |  | *1 (2)* |
| ***TP53 mutation status*** |  |  |  |
| *TP53 M426I* |  |  | *1 (2)* |
| ***Other mutations*** |  |  |  |
| *DPH3 C8T* | *1 (1)* |  |  |
| *NF1 K598E* | *1 (1)* |  |  |
| *NF1 P1851S* | *1 (1)* |  |  |
| *RAC1 P29S* | *2 (2)* |  |  |
| *RPS27 5’UTR* | *1 (1)* |  | *1 (2)* |
| *RQCD1 P131L* | *1 (1)* |  |  |

**BRAF* mutations considered *BRAF* *WT* in relation to qualifying a patient for treatment with BRAF inhibitors
